# Environmental Conditions Influence Allometric Patterns in the Blow Fly, *Chrysomya albiceps*

**DOI:** 10.1673/031.011.13101

**Published:** 2011-10-03

**Authors:** M Battán Horenstein, Av Peretti

**Affiliations:** Consejo Nacional de Investigaciones Científicas y Técnicas; Laboratorio de Biología Reproductiva, Cátedra de Diversidad Animal I, FCEF y N, Universidad Nacional de Córdoba, Av. Vélez Sarsfield 299, 5000, Córdoba, Argentina

**Keywords:** allometry, blow flies, body size, Calliphoridae

## Abstract

The objective of this research was to study variations in allometry of body characters in females and males of two populations of blow flies, *Chrysomya albiceps* (Wiedemann) (Diptera: Calliphoridae), under different environmental conditions to establish patterns of morphological variation. Body size of both males and females in the experimental population was significantly higher than in the individuals of the natural population, indicating an important influence of food on body size. All genitalic and non-genitalic characters in males and females of the two populations showed a trend towards negative allometry rather than isometry. Allometric patterns were modified in both sexes and between populations. The data show generally larger allometric slopes in females than in males. We confirmed that the environmental conditions have an important effect on allometric patterns and body size.

## Introduction

The immature stages of the carrion flies of Calliphoridae, Sarcophagidae, and Muscidae families use different types of organic substrate in decomposition (animal caracasses, dung, etc.) as a source of food. These species are known as “capital breeding insects”, because large body size and reproduction of the adult are completely based on resources— or “capital”—accumulated during larval stages (Jervis and Ferns 2004). Poor nutrition in early life stages has negative effects on many adult life-history characters such as survival, body size, and male characters involved in sexual selection (Jennions and Petrie 1997; Taborsky 2006; Andersen et al. 2010). In carrion flies, natural food of the larval stage is nutritionally rich but ephemeral, limited, and patchy in distribution. As a result, wide variations in the body size and corporal characters size are commonly found in natural populations of adults of these flies in comparison to flies that have been raised on food surpluses since early life stages (Ullyett 1950; Denno and Cothran 1975; Battán Horenstein, personal observations).

Although morphological characters in animals remain more or less constant in both large and small individuals, this may not be a general rule (Cheverud 1982) due to sexual selection pressures that might be acting on them. The variation in the relationship between trait size and body size is know as allometry, and is often described in terms of allometric slope. Isometry is when the trait has the same proportional size in individuals of different body sizes, and negative allometry is when the trait is disproportionately larger in smaller individuals than in larger (Eberhard 2008). Positive allometry is when a trait size increases with body size (Bonduriansky 2007). It has been recognized for a long time that positive allometry is a typical attribute of some sexually selected characters (Eberhard et al. 1998; Peretti et. al. 2001).

Regarding previous studies on allometry in Diptera, Hosken et al. (2005) examined the allometry of genital cerci (sexual trait used by males to grasp females, and possibly to administer “copulatory courtship”), testes, and mandibular palps (nonsexual sensory trait) in *Scatophaga stercoraria* and 12 other scathophagid (Diptera) species. They found that genital cerci are typically negatively allometric, despite sexual selection on this trait. In this respect, is important to point out that male genitalia evolve as a species-specific trait in order to properly fit in female genitalic organs. Additionally, male genitalia show low levels of phenotypic variation (“one size fit all” hypotheses), since genitalic characters are expected to be under strong stabilizing selection (Eberhard et al. 1998). Interestingly, both testis size and mandibular palp length tend to exhibit positive allometry in many species of scathophagid family. However, Borgia (1981) and Partridge et al. (1987) noted in other Diptera species positive allometry of male sexual characters, as well as a strong relationship between the size of these characters and mating success.

Several studies have focused on the effects of environmental quality on developmental stability using fluctuating asymmetry index in different species of flies (Palmer and Strobeck 1992; Lomônaco and Germanos 2001; Soto et al. 2007). Nevertheless, as far as we know, effects of developmental conditions on body morphology and allometric patterns of body characters in Calliphoridae have not yet been explored. The principal aim of this study was examine variations in allometry of body characters in adult females and males of the calliphorid fly, *Chrysomya albiceps* (Wiedemann) (Diptera: Calliphoridae), from two populations raised under different environmental conditions in order to establish major patterns of morphological variation.

## Materials and Methods

### Study materials

*C. albiceps* belongs to the family Calliphoridae. This species was recently introduced into the New World, and since has had a strong impact on the abundance of endemic blowflies, probably as a result of intraguild predatory and aggressive behavior of its larvae (Mariluis and Schnack 1996; Battán Horenstein et al. 2005, 2007, 2010). *C. albiceps* has medical and veterinary importance because it can be mechanical vector of biological pathogens, and can cause myiasis in humans and other vertebrates (Linhares 1981).

Adult males and females of *C. albiceps* (N = 120) were collected using a modified Schoenly (1991) trap during the summer of 2004 in context of a large-scale project studying the development of an ecological community on a pig carcass. These males and females correspond to two pools of individuals. One, referred to as the “natural” pool, consisted of individuals that entered the baited trap in order to either oviposit (females) or mate (both males and females). For this group, nutritional life history was not known. However, it was assumed that these individuals were subjected to different levels of food quality and quantity, as previous studies have demonstrated (Battán Horenstein et al. 2010). The other pool of individuals, referred to as the “experimental” pool, were adult males and females that hatched and emerged inside the trap. These flies were under favorable food quantity conditions in their immature stages.

### Measurement of characters

**Figure 1.**
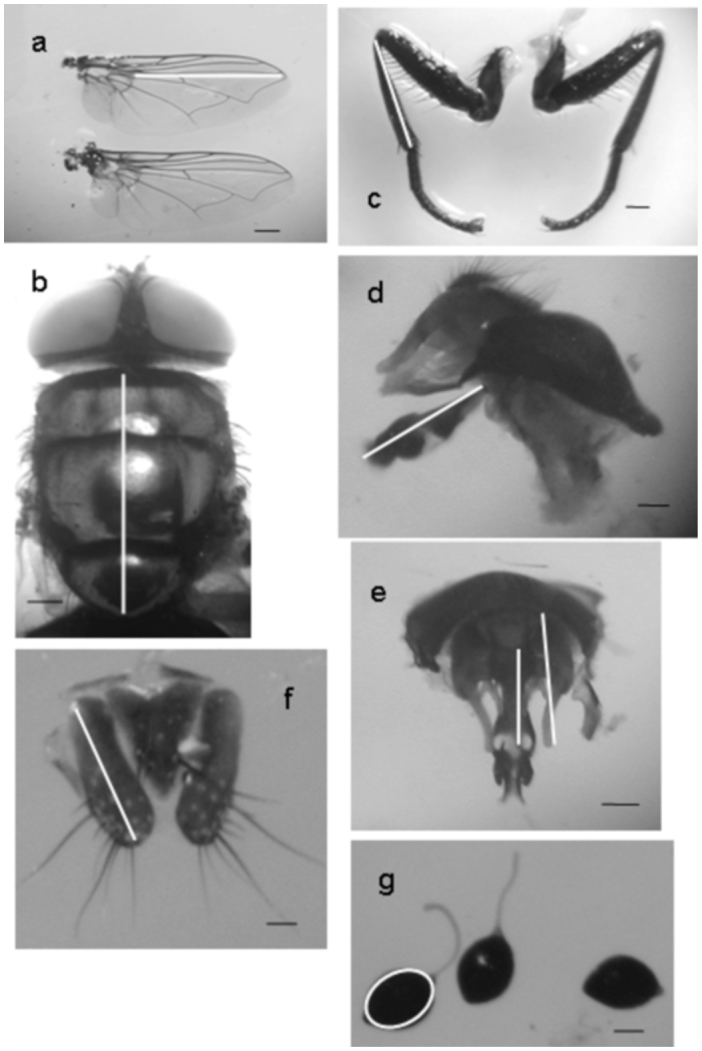
Traits measured in both sexes of each population: a) wing length; b) thorax length; c) fore tibia length; d) aedeagus length; e) male cerci and surstyli length; f) female cerci length; g) spermatheca area. Scale bars: 1 mm. High quality figures are available online.

To measure the different characters, a Leitz stereomicroscope (Leica Microsystems, www.leica-microsystems.com) equipped with an ocular micrometer was used. Thorax length (Figure 1b) was measured in males and females of both populations as an index to estimate body size (Partridge et al. 1987).

Genitalic characters: Male: length of the aedeagus (Figure 1d), cerci and surstyli (lateral lobes) (Figure 1e). Female: length of cerci (Figure 1f) and the area of the three spermathecae (in mm^2^) (Figure 1g).

Non-genitalic characters: For both males and females: wing length (measured from along of the vein R4+5: from the junction with R2+3 to the end of the wing) (Figure 1a) and length of the pro-thoracic tibia (measured from the base to the end in dorsal view) (Figure 1c).

### Statistical analysis

Each trait was measured twice to minimize error. The normality of the variables was tested using the Shapiro-Wilk test. A bilateral *t*-test for independent samples was used to determine intra-sexual, inter-sexual, and inter-population differences in body size. The degree of variability between the genitalic and non-genitalic characters for both sexes in each population was assessed by means of the coefficient of variation (CV) and compared with a Z-test (Zar 1999). Allometry is often described in terms of allometric slope, based on the equation of an ordinary least squares regression (Bonduriansky 2007). Following this criteria used in several studies of allometry (Eberhard 1998; Schmitz et al. 2000; Peretti et al., 2001; Lüpoid et al. 2004; Bonduriansky 2007), ordinary least squares regressions based on log10-transformed data were used to investigate the allometric relationships between body size (thorax length) and each morphological trait. A test of slopes comparison between males and females of two populations was applied to regression lines analysis. Additionally, a one-way ANOVA was made with the residual of the regression lines between the two populations.

## Results

Body size of both males and females from the experimental population was significantly higher than individuals from the natural population, (*p* < 0.01) (Table 1). All the characters measured were significantly larger in the males from the experimental population than in the males from the natural population (*p* < 0.01). The same pattern occurred for females (*p* < 0.01), except that the area of the spermathecae showed no statistically significant differences (Table 1). The females of both populations were significantly larger than males (*p* < 0.01); this difference was weaker between females and males of the experimental population (*p* < 0.05) (Tables 2 and 3). The wings and tibiae in both populations were significantly larger in females than in males, though it should be noted that this difference was not statistically significant in the left tibia in the experimental population.

**Table 1.**
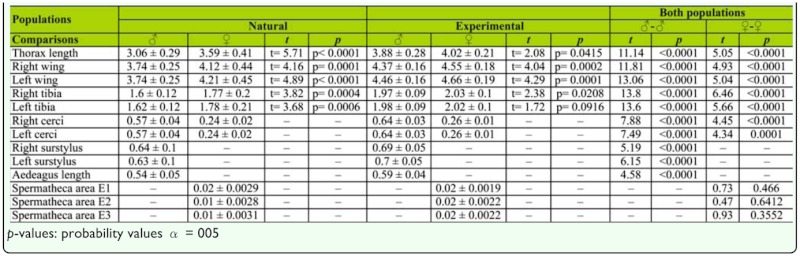
Mean sizes and standard deviations of selected body characters, results of the comparisons (Student t-test) of different body characters between males and females of both populations.

**Table 2.**
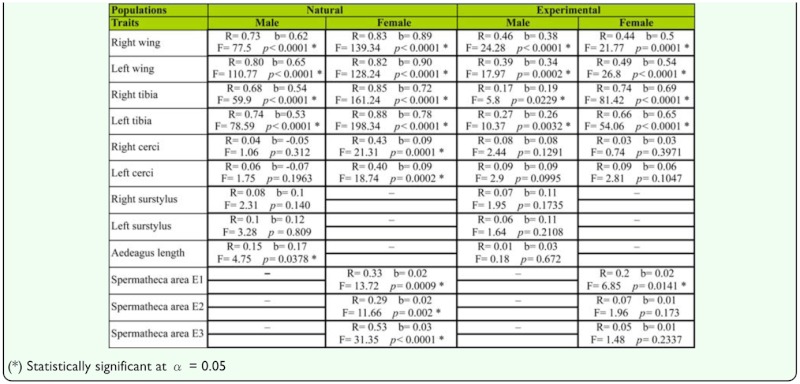
Results of log-log lineal regression analyses.

**Table 3.**
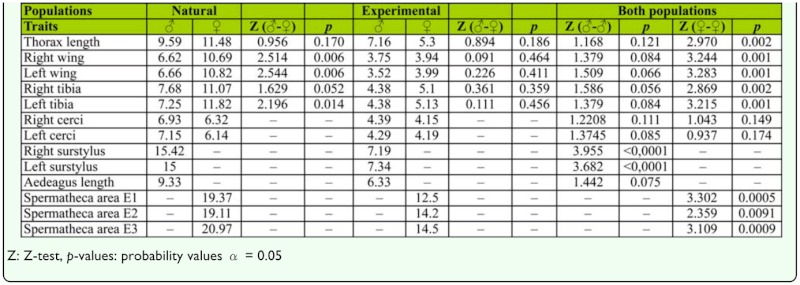
Coefficients of variation (CV) for each variable measured and the results of the comparisons (Z-test) between sexes and populations.

Allometric patterns were modified in both sexes and between populations. The allometric slopes generally were larger in the adults of the natural population than the experimental population. The slopes of allometric regressions of genital and non-genital characters in the natural population was larger in females than in males. The experimental population showed the same pattern for non-genital characters but the inverse pattern for the cercus. The slopes of the regression analyses between males and females for both genitalic and non-genitalic characters were not equal (*t* < 1.671). For females, only the slopes of the wings were parallel, but not coincident and therefore not equal (*t* < 1.671). The one-way ANOVA between residuals of the regression analyses of the two populations showed no statistically significant differences for either genitalic or non-genitalic characters.

Both genitalic and non-genitalic characters of males of the natural population were positively correlated with thorax length, and showed negative allometry, which was statistically significant except in the cerci and surstyli (Table 2). All characters measured in females of this population showed a positive relationship with body size (Table 2). The females showed negative allometry in all of the characters considered. In the males of the experimental population, all the characters considered showed a positive correlation with body size and negative allometry, though statistical significance was only found for the wings and tibia (Table 2). The females of this population showed positive correlation with body size in all characters considered. Negative allometry was observed in these females, but was only statistically significant for wings, tibia, and spermatheca I (Table 2).

The coefficients of variation for the wings and left tibia in the females of the natural population were higher than males (Table 3). In the experimental population, none of the characters measured showed statistically significant results. In the comparisons among males of both populations, coefficient of variation was higher for all characters measured in the natural population compared to the experimental population, though only the surstyli showed statistical significance (Table 3). The same difference was observed in the comparison between females. However, in this case, all characters measured were statistically significant, except the cerci (Table 3).

## Discussion

All of the characters measured were significantly larger in the experimental population than individuals of the same sex in the natural population. This suggests that quality and availability of food affect body size and morphological characters, though feeding regime of the natural animals was not determined in this study. Teder et al. (2008) proposed larger final body size indicates exposure to more favourable conditions during juvenile development. Large females often have greater longevity and higher fecundity, while larger males have enhanced mating success (Bonner 1965; Butler et al. 1984). Male preferences for larger and hence more fecund females have been demonstrated in several species of insects (Hieber and Cohen 1983; Johnson and Hubbell 1984). In the carrion blow fly *Lucilia cuprina*, males prefer females of greater size, which is correlated with greater fertility (Day et al. 1990; Foster et al. 1975).

In this study, while the females under favorable experimental conditions were larger than those in the natural population, both populations showed a negative relationship between body size and spermathecae area. Several factors have been identified that affect adult stage body size, including intraspecific and inter-specific competition, and seasonal changes in food quality during larval stage. The fact that both males and females of the natural population showed greater coefficients of variation (CV) than individuals from the experimental population may indicate a relationship between availability and quality of food and the intraspecific phenotypic variation in adult body size. Our data show that this relationship is negative, in agreement with the suggestions of Teder et al. (2008).

Some structures of male genitalia, such as surstyli, cerci, and aedeagus in both populations showed high CV values compared to other characters measured. Some authors (Møller 1991; Pomiankwoski and Møller 1995) from studies conducted on insects, birds, and mammals proposed that sexually selected characters generally exhibit a high phenotypic variation, which is reflected in higher CV values. In the regression line between body size and each measured trait, the slope values were greater in males from the natural population than in the experimental population. Eberhard et al. (1998) said a high value of CV could be affected both by the dispersion of the values around the regression line and by the slope. The high values of CV observed in this study are likely not due to either the dispersion of the values around the regression line or the slope, since the values of slope were low (mainly in the experimental population). This result could indicate that these characters are being modeled by sexual selection, though it is noteworthy that these characters presented negative allometry. The same pattern was observed for the cerci and spermathecae of females in both populations.

These results are in concordance with those observed in other species by Eberhard (2009) and Bernstein and Bernstein (2002). Recent studies show that the allometric slopes of genitalic characters are lower than those of other body characters, giving rise to the “one size fits all” hypothesis (Eberhard et al. 1998). This hypothesis suggests that sexual selection favors males with genitalia appropriate to stimulate any female during the mating season (Eberhard et al. 1998; Bernstein and Bernstein 2002). Eberhard (2009) proposed that the male genitalia could be subject to other mixed pressures, such as natural and sexual selection, which could be implied in the modeling of genitalic characters. In contrast, some studies suggest that the morphology of the male genitalia could be linked to mating success, indicating that specific associated structures would be used by females to test male quality more than genitalia size. In these cases, the allometric slopes observed are positive (Arnqvist and Danielsson 1999; House and Simmons 2002). In addition, it is important to point out that in studies of sexual selection in Diptera, Borgia (1981) and Partridge et al. (1987) observed positive allometry in male genitalia characters (aedeagus and cerci), and a strong relationship between their size and mating success.

The non-genitalic characters measured in this study also showed negative allometry. Bonduriansky (2007) and other authors proposed that sexual selection almost invariably leads to the evolution of positive allometry in secondary sexual characters. Taking this into account, our results indicate that these characters would be subjected to sexual selection. Another possibility could be that sexual selection operates differentially on these characters, perhaps by stabilizing selection, in order to keep the size of genital characters stable, and to eliminate individuals with traits that do not allow them to effectively contribute to mating and sperm transfer.

**Figure 2.**
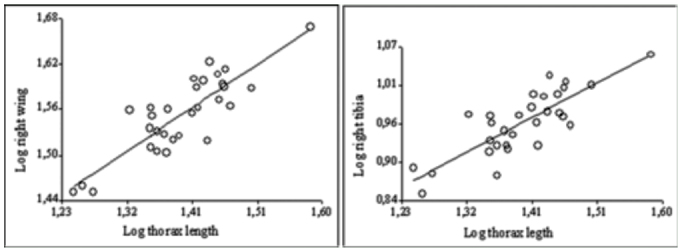
Scatter plots of the significant relationships between body size (thorax length) and non-genitalic characters in males of the natural population. Both side of each trait show the same pattern. High quality figures are available online.

**Figure 3.**
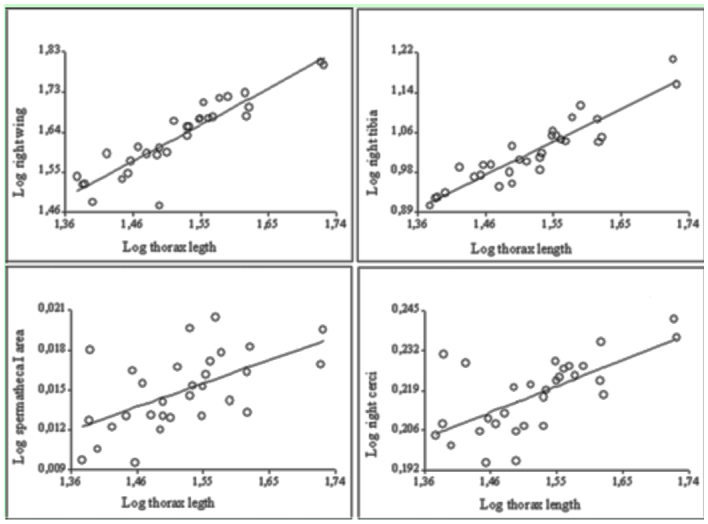
Scatter plots of the significant relationships between body size (thorax length) and genitalic and non-genitalic characters in females of the natural population. Both side of each trait and the three spermathecae show the same pattern. High quality figures are available online.

**Figure 4.**
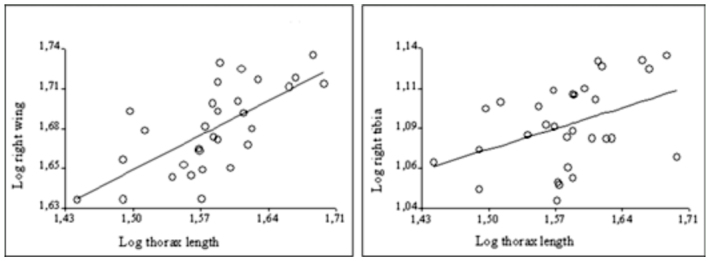
Scatter plots of the significant relationships between body size (thorax length) and non-genitalic characters in males of the experimental population. Both side of each trait show the same pattern. High quality figures are available online.

**Figure 5.**
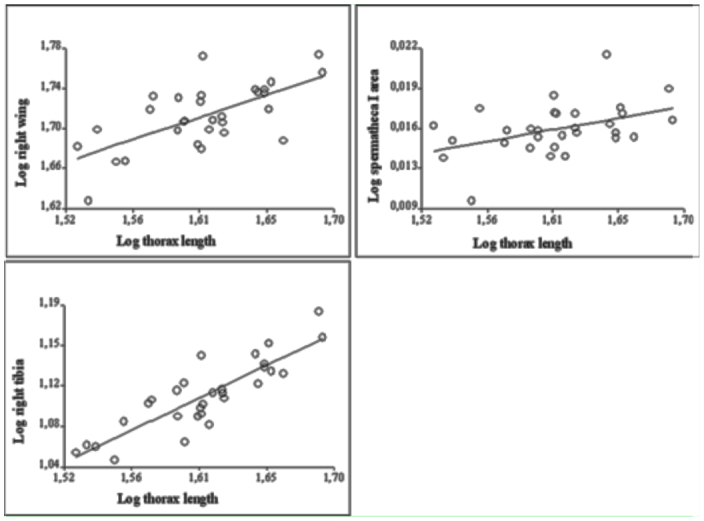
Scatter plots of the significant relationships between body size (thorax length) and genitalic and non-genitalic characters in females of the experimental population. Both side of each trait show the same pattern but only spermatheca I was statistically significant. High quality figures are available online.
